# Islet Regeneration and Pancreatic Duct Glands in Human and Experimental Diabetes

**DOI:** 10.3389/fcell.2022.814165

**Published:** 2022-02-04

**Authors:** Diletta Overi, Guido Carpino, Marta Moretti, Antonio Franchitto, Lorenzo Nevi, Paolo Onori, Enrico De Smaele, Luca Federici, Daniele Santorelli, Marella Maroder, Lola M. Reid, Vincenzo Cardinale, Domenico Alvaro, Eugenio Gaudio

**Affiliations:** ^1^ Department of Anatomical, Histological, Forensic Medicine and Orthopedic Sciences, Sapienza University of Rome, Rome, Italy; ^2^ Department of Movement, Human and Health Sciences, Division of Health Sciences, University of Rome “Foro Italico”, Rome, Italy; ^3^ Department of Experimental Medicine, Sapienza University of Rome, Rome, Italy; ^4^ Department of Biosciences, University of Milan, Milan, Italy; ^5^ CAST Center for Advanced Studies and Technology and Department of Innovative Technologies in Medicine and Odontoiatry, University “G. D’Annunzio” of Chieti-Pescara, Chieti, Italy; ^6^ Department of Biochemical Sciences “Rossi Fanelli”, Sapienza University of Rome, Rome, Italy; ^7^ Department of Molecular Medicine, Sapienza University of Rome, Rome, Italy; ^8^ Departments of Cell Biology and Physiology, Program in Molecular Biology and Biotechnology, UNC School of Medicine, University of North Carolina, Chapel Hill, NC, United States; ^9^ Department of Medico-Surgical Sciences and Biotechnologies, Sapienza University of Rome, Latina, Italy; ^10^ Department of Translational and Precision Medicine, Sapienza University of Rome, Rome, Italy

**Keywords:** stem/progenitor cells, endocrine pancreas, streptozotocin, Pdx1, insulin

## Abstract

Contrasting evidence is present regarding the contribution of stem/progenitor cell populations to pancreatic regeneration in diabetes. Interestingly, a cell compartment with stem/progenitor cell features has been identified in the pancreatic duct glands (PDGs). The aims of the present study were to evaluate pancreatic islet injury and regeneration, and the participation of the PDG compartment in type 2 diabetic mellitus (T2DM) and in an experimental model of diabetes. Human pancreata were obtained from normal (N = 5) or T2DM (N = 10) cadaveric organ donors. Experimental diabetes was generated in mice by intraperitoneal injection of 150 mg/kg of streptozotocin (STZ, N = 10); N = 10 STZ mice also received daily intraperitoneal injections of 100 µg of human recombinant PDX1 peptide (STZ + PDX1). Samples were examined by immunohistochemistry/immunofluorescence or RT-qPCR. Serum glucose and c-peptide levels were measured in mice. Islets in T2DM patients showed β-cell loss, signs of injury and proliferation, and a higher proportion of central islets. PDGs in T2DM patients had a higher percentage of proliferating and insulin^+^ or glucagon^+^ cells compared to controls; pancreatic islets could be observed within pancreatic duct walls of T2DM patients. STZ mice were characterized by reduced islet area compared to controls. PDX1 treatment increased islet area and the percentage of central islets compared to untreated STZ mice but did not revert diabetes. In conclusion, T2DM patients show signs of pancreatic islet regeneration and involvement of the PDG niche. PDX1 administration could support increased endocrine pancreatic regeneration in STZ. These findings contribute to defining the role and participation of stem/progenitor cell compartments within the pancreas.

## Introduction

Diabetes mellitus comprises metabolic diseases characterized by hyperglycemia. Type 1 diabetes mellitus (T1DM) is caused by an autoimmune destruction of pancreatic β-cells, while type 2 diabetes mellitus (T2DM) develops due to insulin resistance and can progress towards β-cell dysfunction (Mathieu et al., 2021). In these patients, regenerative processes can occur, attempting to compensate for the loss of β-cells (Yoneda et al., 2013). Therefore, the identification and characterization of regenerative trajectories within the pancreas could provide insight for the development of novel therapeutic strategies in diabetes treatment.

In the past years, evidence has emerged challenging the hypothesis of the presence of progenitor cell populations within the pancreas participating in islet regeneration; in particular, lineage tracing-based studies have indicated β-cell renewal to be sustained by mature cell replication more than progenitor cell commitment (Domínguez-Bendala et al., 2019). Interestingly, remnants of hepato-bilio-pancreatic precursors have been identified in the biliary tree and in the pancreatic duct system (Cardinale et al., 2012). In particular, the biliary tree stem/progenitor cells (BTSCs) have been identified within the peribiliary glands (PBGs) of the larger intrahepatic and extrahepatic bile ducts, and represent a multipotent stem/progenitor cell compartment. Their capabilities to differentiate towards mature endocrine pancreatic cells have been evaluated both *in vitro* and *in vivo* (Lanzoni et al., 2016). In particular, it has been shown how pancreatic and duodenal homeobox 1 (PDX1) can modulate the balanced differentiation of progenitor/stem cells towards endocrine pancreas commitment rather than towards the biliary fate (Cardinale et al., 2015). We have previously expressed human PDX1 sequence in *E. Coli* and tested *in vitro* the effects of the recombinant PDX1 protein on inducing differentiation toward pancreatic islet cells in BTSCs. We observed how PDX1 can trigger the expression of both intermediate and mature stage β-cell differentiation markers in BTSCs (Cardinale et al., 2015).

In parallel to the PBGs, the pancreatic duct system harbors similar glandular compartments: the pancreatic duct glands (PDGs) are tubulo-acinar glands located within the lamina propria of main pancreatic ducts and, occasionally, large interlobular ducts (Carpino et al., 2016a). PDGs have been shown to harbor a niche of committed precursors towards pancreatic fates (Carpino et al., 2016a). However, the response of this cellular compartment in diabetes has not been investigated yet.

Therefore, the aims of the present study were: 1) to evaluate pancreatic islet injury and phenotype in T2DM patients and in an experimental model of diabetes; 2) to describe the modifications of the PDG compartment in type 2 diabetic patients and in an experimental model of diabetes; 3) to test the possible effects of recombinant PDX1 administration on pancreatic islets and PDG in an experimental model of diabetes.

## Materials and Methods

### Human Samples

Human pancreata were obtained from cadaveric donors (N = 15) from the surgical department of Policlinico Umberto I, Sapienza University of Rome, Italy. Based on anamnestic and serological data, samples were divided into normal (N = 5) or T2DM (N = 10). Informed consent was obtained from next of kin for use of the tissues for research purposes. Study protocols received Institutional Review Board approval from Policlinico Umberto I. Pancreas and duodenum were obtained *en bloc* from organ transplantation procedures. For each case, samples were taken at the level of the main pancreatic duct prior to merging with the choledocus, and at the different levels of the pancreatic body and tail.

### Histomorphology, Immunohistochemistry, and Immunofluorescence

Specimens were fixed in 10% buffered formalin and embedded in paraffin, and 3–5 μm sections were obtained and processed for hematoxylin and eosin (H&E). For immunohistochemistry, endogenous peroxidase activity was blocked by a 30-min incubation in 2.5% hydrogen peroxide. Sections were incubated overnight at 4°C with primary antibodies (listed in Supplementary Table S1). Then, samples incubated for 20 min at room temperature with secondary biotinylated antibody, and then with streptavidin-horseradish peroxidase (LSAB+, Dako, Glostrup, Denmark, code: K0690). Diaminobenzidine (Dako, Glostrup, Denmark, code: K3468) was used as substrate, and sections were counterstained with hematoxylin. Sections were examined in a coded fashion by Leica Microsystems DM4500B Light and Fluorescence Microscopy (Weltzlar, Germany), equipped with a Jenoptik Prog Res C10 Plus Videocam (Jena, Germany).

For immunofluorescence (IF), non-specific protein binding was blocked by 5% normal goat serum. Specimens were incubated with primary antibodies overnight; then, samples were washed and incubated for 1 h with labeled isotype-specific secondary antibodies (AlexaFluor^®^, Invitrogen, Life Technologies Ltd., Paisley, United Kingdom) and counterstained with 4,6-diamidino-2-phenylindole (DAPI) for visualization of cell nuclei. To perform double immunostaining with two primary antibodies from the same host species, we followed a 3-step protocol: sections were incubated with the first primary antibody; then, a secondary fluorescent antibody was applied; finally, the second primary antibody was pre-labeled with a fluorophore using the APEX-594 labeling Kit (Invitrogen) and applied to the section.

For all immunoreactions, negative controls (the primary antibody was replaced with pre-immune serum) were also included.

Sections were examined in a coded fashion by Leica Microsystems DM4500B Light and Fluorescence Microscopy (Weltzlar, Germany), equipped with a Jenoptik Prog Res C10 Plus Videocam (Jena, Germany). Immunofluorescence stains were also analyzed by Confocal Microscopy (Leica TCS-SP2). Slides were further scanned by a digital scanner (Aperio Scanscope CS and FL Systems, Aperio Digital Pathology, Leica Biosystems, Milan, Italy) and processed by ImageScope.

The area of pancreas occupied by the islets of Langerhans and islet’s size were evaluated on H&E slides by ImageScope. Islets were considered as “central” or “peripheral” based on their position with respect to the pancreatic lobule and duct system: central islet are typically located close to interlobular septa, connected to a clearly-defined pancreatic inter/intralobular duct and in continuity with duct’s surrounding stroma; peripheral islets are located in the middle of pancreatic lobule without connection with inter/intralobular duct stroma (Merkwitz et al., 2013). Islet composition was evaluated by counting positive cells within islets. Moreover, the expression of nuclear antigens was automatically calculated by a specific algorithm on selected areas and expressed as a percentage of positive cells.

### Streptozotocin (STZ)-Induced Diabetic Mice and PDX1 Treatment

Male NOD/SCIDgamma (NSG) mice (N = 25) were purchased from Charles River (Calco, Milan, Italy). Mice were housed in a dedicated, pathogen-free barrier facility at the Sapienza University of Rome in compliance with Italian regulations. Mice were kept in a room with specific pathogen-free standards maintained at a temperature of 23 ± 1°C and 50 ± 10% relative humidity, with food and water available *ad libitum*. The animal room was on a 12:12 h light:dark cycle. Mice were individually identified by ear punching.

Type 1 diabetes mellitus was induced by a single intraperitoneal injection of a single dose of 150 mg/kg (N = 10) of STZ. Moreover, N = 10 additional STZ mice were treated with daily intraperitoneal injections of 100 µg of human recombinant PDX1 peptide (Cardinale et al., 2015). Mice in the STZ group were injected with saline solution. N = 5 mice were included as controls and did not receive STZ or PDX1. Animals that reached stable glucose levels >300 mg/dl were considered as diabetic (Leiter and Schile, 2013). The study was conducted on NSG mice in order to avoid a possible immune reaction of the host against the human PDX1.

Treatment with PDX1 was initiated 48 h after STZ injection and confirmation of stable serum glucose levels >300 mg/dl. To prevent mortality due to the hypoglycemia caused by massive insulin release after STZ-induced pancreatic islet damage, animals were treated with water supplemented with 10% sucrose for 48 h after STZ administration. Glucose levels were measured every 3 days by AlphaTRAK glucometer with strips (Abbott). Pancreatic tissue samples were obtained at sacrifice. Tissues were processed for histology, immunohistochemistry and immunofluorescence, or frozen for RT-qPCR analysis. All animal experiments were approved by the institutional animal care and use committee of Sapienza University of Rome and by the Italian Ministry of Health.

### PDX1 Production and Purification

Recombinant PDX1 was obtained in the form of a fusion protein by linking 6His-tag to the N-terminus of the amino acid sequence. Full-length DNA coding sequence for human PDX1 (852 bp coding for 283 aa) adapted for heterologous expression in *E. Coli* was provided by GenScript United States Inc. (Piscataway, NJ). The sequence was amplified by PCR using primers 5′-TAT​CAT​ATG​AAC​GGT​GAA​GAA​CAG​TAC​TAC-3′ and 5′-ATA​CTC​GAG​CTA​ACG​TGG​TTC​TTG​CGG​ACG​GC-3’. After digestion with NdeI and BamHI, the amplicon was ligated into pET-28a expression vector (Novagen-Merck, Darmstadt, Germany), yielding pET-PDX1 plasmid. This construct was used to transform the BL21 (DE3) *E. Coli* strain (Invitrogen). BL 21 (DE3) cells were grown in Luria Bertani (LB) medium containing 34 μg/ml Kanamycin and at 37°C until the OD_600_ reached 0.6; then, protein expression was induced with 1 mM IPTG. After induction, cells were grown at 21°C overnight and then collected by centrifugation. For PDX1 purification, the cell pellet was resuspended in 20 mM Tris-HCl pH 8.0, 0.2 U/mL of Benzonase nuclease (Sigma-Aldrich), 5 mM MgCl2, protease inhibitor tablet (Complete EDTA-free, Roche), and glycerol 10%, sonicated and centrifuged. After addition of 500 mM NaCl and 25 mM imidazole, the soluble fraction was loaded on a 5 ml HisTrap FF (GE Healthcare) pre-equilibrated with resuspension buffer. The protein was eluted with an imidazole gradient (20 mM–1 M imidazole in buffered Tris-HCl (pH 8.0, NaCl 500 mM, glycerol 10%) and then fractions containing PDX1 protein were analyzed by SDS-PAGE. A Sephadex G-25 column (GE Heathcare) was employed to remove imidazole and to exchange buffer with PBS. Mass spectrometry analyses were performed after tryptic digestion of the band of 43 kDa isolated by Coomassie blue stained gel. Mass spectra were acquired by Ultraflex III MALDI-TOF-TOF instrument (Bruker-Daltonics, Bremen, Germany), and peptide sites were searched in the NCBI database by MASCOT search engine.

### RT-qPCR Analysis and ELISA Assay

Total RNA was extracted by the procedures of Chomczynski and Sacchi (Chomczynski and Sacchi, 2006). RNA quality and quantity was evaluated with the Experion Automated Electrophoresis System. RNA equipped with the RNA StSens Analysis Chip (Bio-Rad Laboratories, Hercules, CA). RNA was extracted by TRIZOL reagent (Life Technologies, Rockville, MD; Cat# 15,596-026) according to manufacturer’s instructions. One µg of RNA was retrotranscribed using High Capacity cDNA Reverse Transcription Kit (Applied Biosystems, Life Technologies, Paisley, United Kingdom: code 4368814), and cDNA was amplified using SensiMix SYBR kit (Bioline, London, United Kingdom: code QT605-05) according to manufacturer’s instructions. The primers used are listed in Supplementary Table S2. The expression of the gene of interest was calculated by the ratio of the concentrations of the gene of interest and the reference gene 18 s.

Serum mouse C-peptide levels in response to glucose administration were measured by ELISA assay. Mice were fasted overnight, and 30% dextrose was injected intraperitoneally at 2 g/kg body weight. Sixty minutes after the glucose injection, 80 μl of blood were collected into heparinized micro-hematocrit capillary tubes (Fisherbrand) and prepared serum samples were subjected to assays for mouse C-peptide. The Ultrasensitive Mouse C-peptide ELISA kit (ALPCO, Catalog Number 80-CPTMS-E01) was used according to manufacturer’s instructions.

### Statistical Analysis

Continuous data are presented as mean ± SD. Student’s t-test or Mann–Whitney *U*-test were used to determine differences between groups for normally- or not normally-distributed data, respectively. A One-way ANOVA or Kruskal–Wallis H test were used to calculate differences between three groups. A *p*-value <0.05 was considered statistically significant. Analyses were performed using IBM SPSS software (SPSS Inc., United States).

## Results

### Islet of Langerhans Modifications in Human Diabetes

**FIGURE 1:**
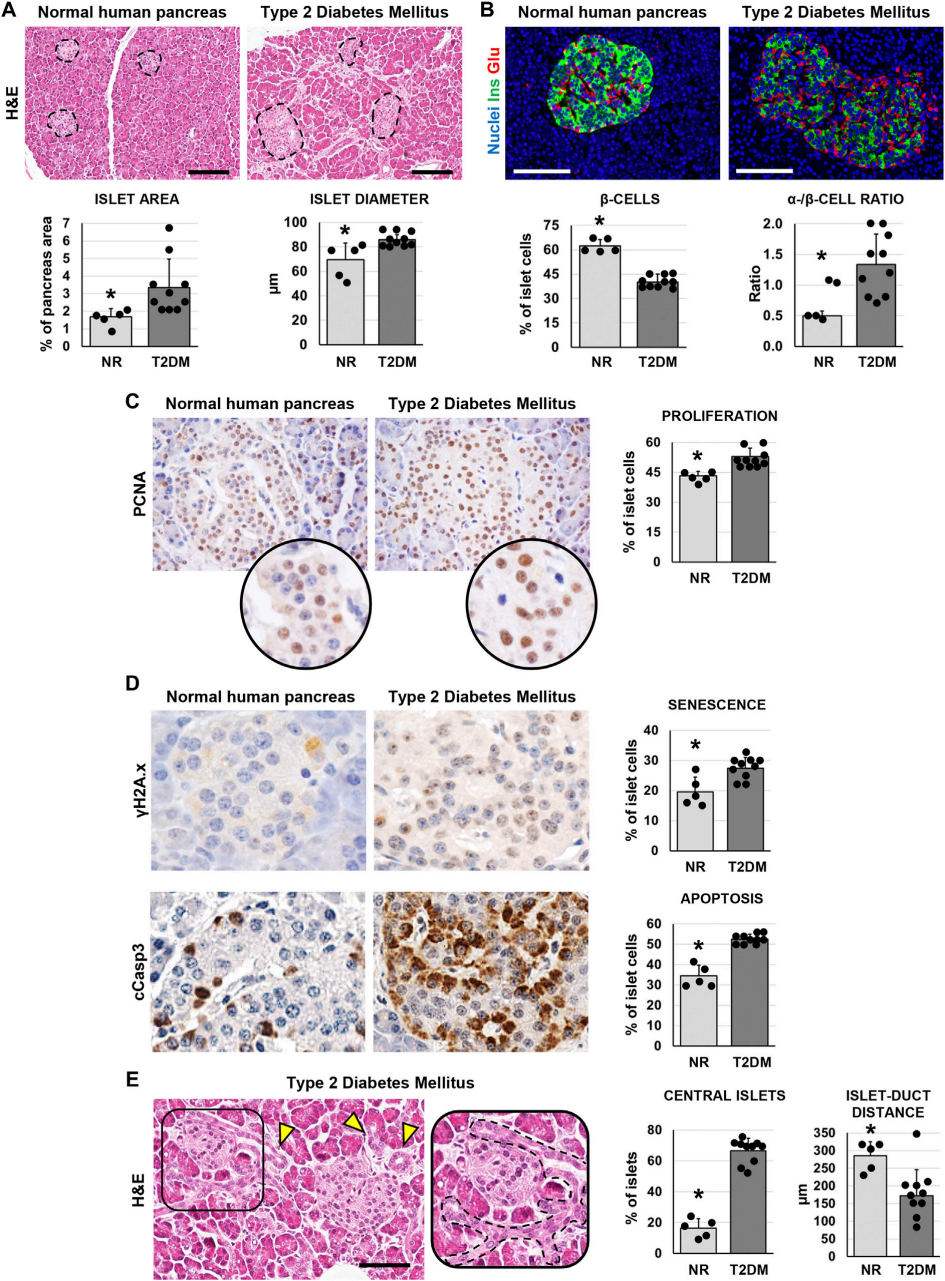
Pancreatic islet histology and phenotype in normal (NR) human pancreas and in type 2 diabetes mellitus (T2DM) pancreas samples. **(A)** Hematoxylin and eosin (H&E) stain. T2DM patients showed higher islet area and islet size compared to normal samples. Dotted lines individuate pancreatic islets. Histograms show means and standard deviation (SD) for islet area and diameter. Scale bar: 150 μm. **(B)** Double immunofluorescence for insulin (green) and glucagon (red). Pancreatic islets in T2DM patients were characterized by a lower β-cell percentage and by a higher α-/β-cell ratio compared to normal pancreata. Histograms show means and SD for β-cell percentages and for the α-/β-cell ratio. Scale bar: 100 μm. Nuclei are displayed in blue (DAPI staining). **(C)** Immunohistochemistry for proliferating cell nuclear antigen (PCNA). T2DM patients showed increased percentage of proliferating PCNA + cells within islets compared to normal pancreata. Original magnification: 40x. Areas in the circle are magnifications of the images above. The histogram shows means and SD for the percentage of proliferating cells. **(D)** Immunohistochemistry for γH2A.x (upper panels) and cleaved caspase 3 (cCasp3, lower panels). Pancreatic islets in diabetic patients were characterized by a higher expression of senescence marker γH2A.x and apoptosis marker cCasp3 compared to normal pancreata. Histograms show means and standard deviation for the percentage of positive cells. Original magnification: 40x. **(E)** H&E stain on pancreata from T2DM patients. Pancreata from T2DM patients were characterized by a higher percentage of central islets compared to normal ones. Arrowheads indicate pancreatic ducts. The area in the box is magnified on the right; dotted line individuates a pancreatic duct branch surrounding an islet. Scale bar: 75 μm. The histogram shows means and SD for the percentage of central islets and the average distance between islets and neighboring ducts. * = p< 0.05 versus T2DM.

In pancreata obtained from patients affected by T2DM, the area occupied by islets was significantly higher (3.3 ± 1.7%) compared to normal ones (1.8 ± 0.5%; *p* < 0.05; Figure 1A) In parallel, pancreatic islets in T2DM samples were larger (86.0 ± 4.4 µm) compared to the islets in normal pancreata (69.8 ± 13.5µm; *p* < 0.05; Figure 1A). However, when pancreatic islet composition was investigated (Figure 1B), islets in T2DM were characterized by a lower percentage of β-cells (40.2 ± 4.7%) and by a higher percentage of α-cells (55.0 ± 9.6%) compared to normal pancreata (62.2 ± 4.4% and 31.5 ± 6.1%, respectively; *p* < 0.001 and *p* < 0.01); therefore, T2DM was characterized by a higher α-/β-cell ratio (1.35 ± 0.49) compared to islets in normal pancreata (0.5 ± 0.08; *p* < 0.01).

We then performed immunohistochemical analysis to evaluate cell proliferation (by proliferating cell nuclear antigen—PCNA), senescence (by γH2A.x) and apoptosis (by cleaved caspase 3—cCasp3) in pancreatic islets. The percentage of PCNA + islet cells was higher in T2DM (52.9 ± 4.2%) compared to normal pancreata (43.2 ± 2.3%; *p* < 0.01; Figure 1C); however, cells within pancreatic islets in T2DM patients also showed an increase of γH2A.x (27.5 ± 3.5%) and cCasp3+ (52.6 ± 2.3%) expression compared to normal pancreatic islets (19.6 ± 4.9% and 34.4 ± 5.4%, respectively; *p* < 0.05 and *p* < 0.001; Figure 1D).

Interestingly, when we evaluated the localization of pancreatic islets, we observed a higher proportion of central islets in T2DM patient pancreata (66.7 ± 7.9%) compared to normal ones (16.4 ± 6.3%; *p* < 0.001; Figure 1E). Pancreatic islets were less distant from neighboring ducts in T2DM (172.0 ± 74.5 µm) compared to normal subject pancreata (285.4 ± 40.33 µm; *p* < 0.01).

### Pancreatic Duct Glands (PDGs) in Human Type 2 Diabetes Mellitus

**FIGURE 2:**
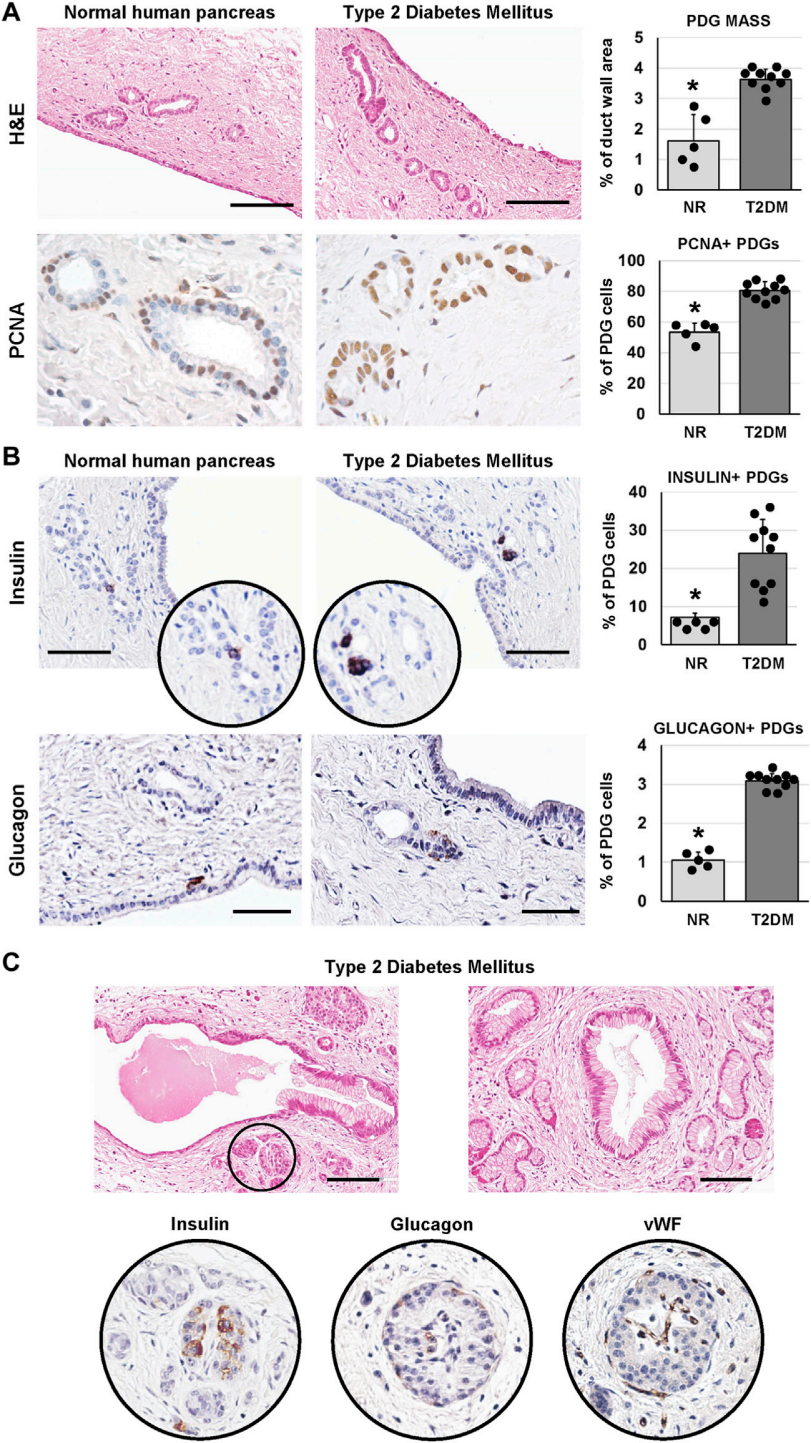
Pancreatic duct glands (PDGs) in normal (NR) human pancreas and in type 2 diabetes mellitus (T2DM) pancreas samples. **(A)** Hematoxylin and eosin (H&E) stain (upper panels) and immunohistochemistry for proliferating cell nuclear antigen (PCNA, lower panels). Pancreatic ducts in T2DM patients were characterized by a higher PDG mass and by a higher expression of PCNA within PDGs compared to normal ducts. Histograms show means and standard deviation (SD) for the percentage of duct wall area occupied by PDGs and for the percentage of PCNA + PDG cells. Scale bar for H&E: 100 μm. Original magnification for PCNA: 40x. **(B)** Immunohistochemistry for insulin (upper panels) and glucagon (lower panels). T2DM patients showed a higher percentage of insulin+ and glucagon + cells within PDGs compared to normal ones. Histograms show means and SD for the percentage of positive cells. Scale bar: 75 μm (insulin) and 50 μm (glucagon). **(C)** H&E stain on T2DM samples shows pancreatic ducts with dysplastic lesions of surface epithelium and PDGs and the presence of pancreatic islets among PDGs (circle). Seriated sections of the same area show that islets in pancreatic ducts express insulin, glucagon and are vascularized. Scale bar: 100 μm * = p< 0.05 versus T2DM.

We investigated whether the PDG compartment could be modified in T2DM-affected pancreata. Interestingly, main pancreatic duct samples obtained from T2DM patients were characterized by a higher area occupied by PDGs in the duct wall (i.e. PDG mass; 3.6 ± 0.3%) compared to normal samples (1.6 ± 0.9%; *p* < 0.01; Figure 2A). Accordingly, an increased percentage of PCNA + cells was observed in PDGs of T2DM patients (80.7 ± 5.8%) compared to normal ones (53.4 ± 5.8%; *p* < 0.001; Figure 2A).

As in our previous study (Carpino et al., 2016a), cells within PDGs expressing insulin or glucagon could be observed (Figure 2B). Interestingly, PDGs in diabetic pancreata were characterized by a higher percentage of insulin+ (23.9 ± 8.9%) and glucagon+ (3.1 ± 0.2%) cells as compared to normal organs (7.2 ± 1.1% and 1.1 ± 0.2%, respectively; *p* < 0.05).

Uniquely, large islet-like structures could be found within main pancreatic duct walls in T2DM samples; these islets showed positivity for insulin and glucagon and were characterized by the presence of blood vessels within the islet (Figure 2C). Of note, main pancreatic duct in T2DM but not in normal samples, were also characterized by the presence of dysplastic lesions of the surface epithelium and PDGs (chi-squared test *p* < 0.05; Figure 2C).

### Pancreatic Islet Injury and PDG Activation in Murine Streptozotocin-Induced Diabetes

**FIGURE 3:**
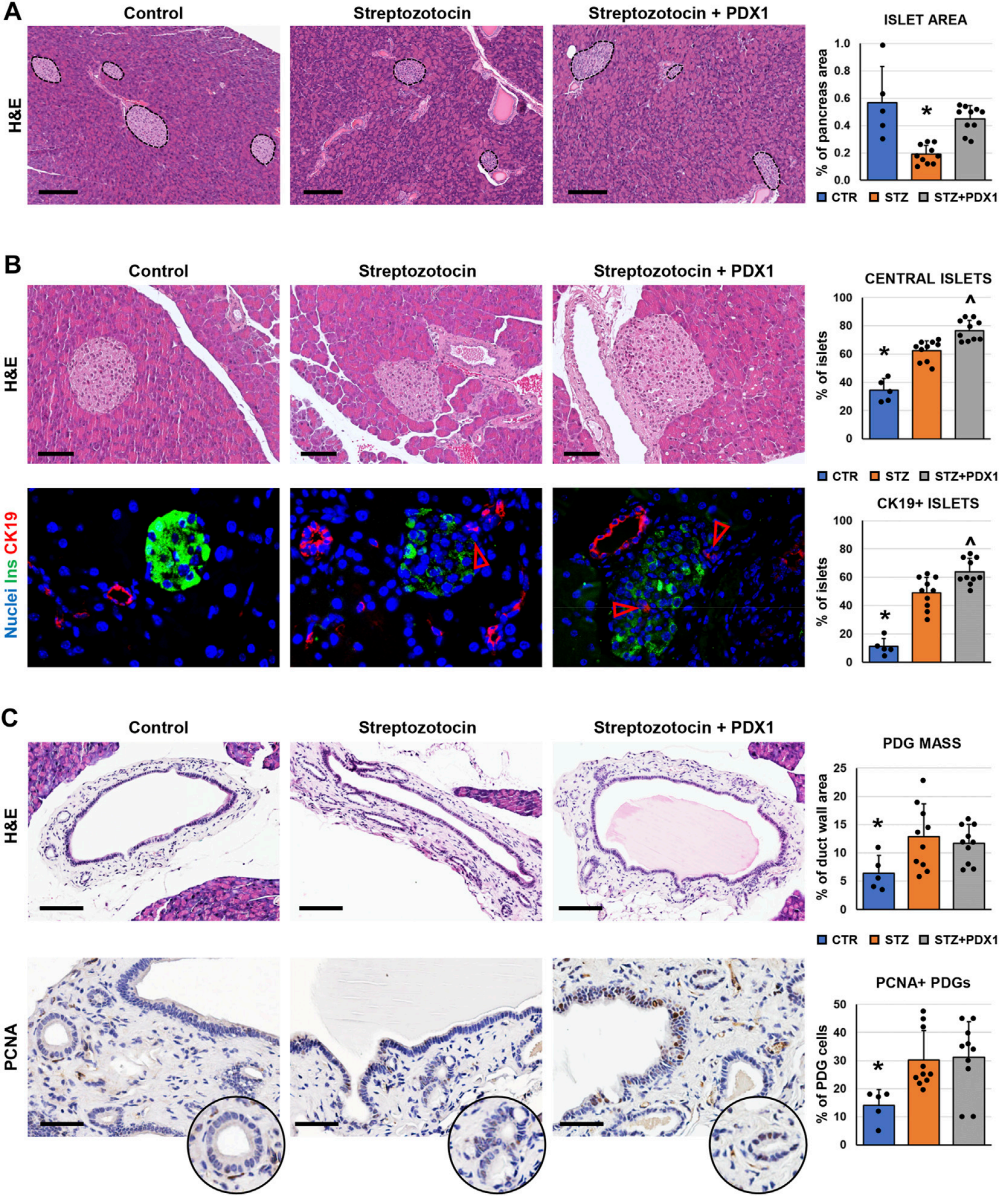
Pancreatic islets and pancreatic duct glands (PDGs) in control mice, streptozotocin (STZ)-treated mice and STZ mice treated with PDX1. **(A)** Hematoxylin and eosin (H&E) stain on pancreas samples. STZ treated mice were characterized by a lower pancreatic islet area compared to controls; STZ + PDX1 mice showed a higher islet area compared to STZ, without significant differences compared to controls. Dotted lines individuate pancreatic islets. Histogram shows means and standard deviation (SD) for pancreatic area percentage. Scale bar: 150 μm. **(B)** H&E stain (upper panels) and double immunofluorescence for insulin (ins, in green) and cytokeratin 19 (CK19, in red) on pancreas samples. STZ-treated mice show a higher percentage of central islets compared to controls; moreover, STZ + PDX1 mice showed a significantly higher percentage of central islets compared to STZ and control mice. Scale bar in H&E: 75 μm. In immunofluorescence image, arrowheads indicate small CK19 + cells within islets. Nuclei are displayed in blue (DAPI staining). Original magnification: 40x. Histograms show means and SD for the percentage of central islets and islets containing CK19 + cells. **(C)** H&E stain and immunohistochemistry for proliferating cell nuclear antigen (PCNA) on pancreatic ducts. STZ- and STZ + PDX1-treated mice show a higher PDG mass and percentage of PCNA + cells within PDGs compared to controls. Histograms show means and SD for the percentage of duct wall area occupied by PDGs and for the percentage of PCNA + PDG cells. Scale bar: 100 μm (H&E) and 50 μm (PCNA). * = p< 0.05 versus other groups; ^ = p< 0.05 versus STZ group.

We further investigated pancreatic islet morphology after injury in a murine model of diabetes, the STZ mice. We first examined the pathological modification of islets in mice, and we observed that the area of pancreatic islets was lower in STZ mice (0.19 ± 0.07%) compared to controls (0.57 ± 0.27%; *p* < 0.05; Figure 3A). Moreover, pancreatic islets were smaller in STZ mice (diameter: 60.7 ± 15.6 µm) compared to control ones (88.6 ± 19.3µm; *p* < 0.05). Interestingly, the area of pancreatic islets was higher in STZ mice treated with PDX1 (0.45 ± 0.10%) than in untreated STZ mice (*p* < 0.01); no significant difference was observed between STZ + PDX1-treated and control mice.

When the anatomical location of islets was studied (Figure 3B), the percentage of central islets was higher in mice treated with STZ (62.3 ± 7.1%) compared to control mice (34.5 ± 8.1%; *p* < 0.001). Furthermore, STZ + PDX1-treated mice were characterized by a higher percentage of central islets (76.5 ± 7.3%) compared to untreated STZ (*p* = 0.03) and control mice (*p* < 0.001). Islets in STZ + PDX1-treated mice showed a closer proximity to ducts (59.0 ± 13.5 μm) compared to STZ (79.3 ± 22.9 μm; *p* < 0.05) and control mice (154.8 ± 7.8 μm *p* < 0.05). Ductular CK19 + cells were found inside pancreatic islets (Figure 3B), and the percentage of islets with CK19 + cells was higher in STZ + PDX1 (63.7 ± 9.7) compared to STZ (49.0 ± 10.9; *p* < 0.05) and control mice (11.2 ± 5.5; *p* < 0.05).

We then investigated the modifications of the PDG cell compartment in STZ mice (Figure 3C). Interestingly, both STZ and STZ + PDX1 mice were characterized by a higher PDG mass (12.8 ± 5.8% and 11.7 ± 3.3%, respectively) compared to control ones (6.4 ± 3.1%; *p* < 0.05). Accordingly, PDG cells in STZ and STZ + PDX1 mice were characterized by a higher expression of PCNA (30.1 ± 10.5% and 31.2 ± 12.6%, respectively) compared to controls (14.1 ± 5.7%; *p* < 0.05). No significant differences were observed between STZ and STZ + PDX1 mice in term of PDG area and PCNA expression in PDG cells.

### Islet Phenotype and Glycemic Profile in Murine Streptozotocin-Induced Diabetes

**FIGURE 4:**
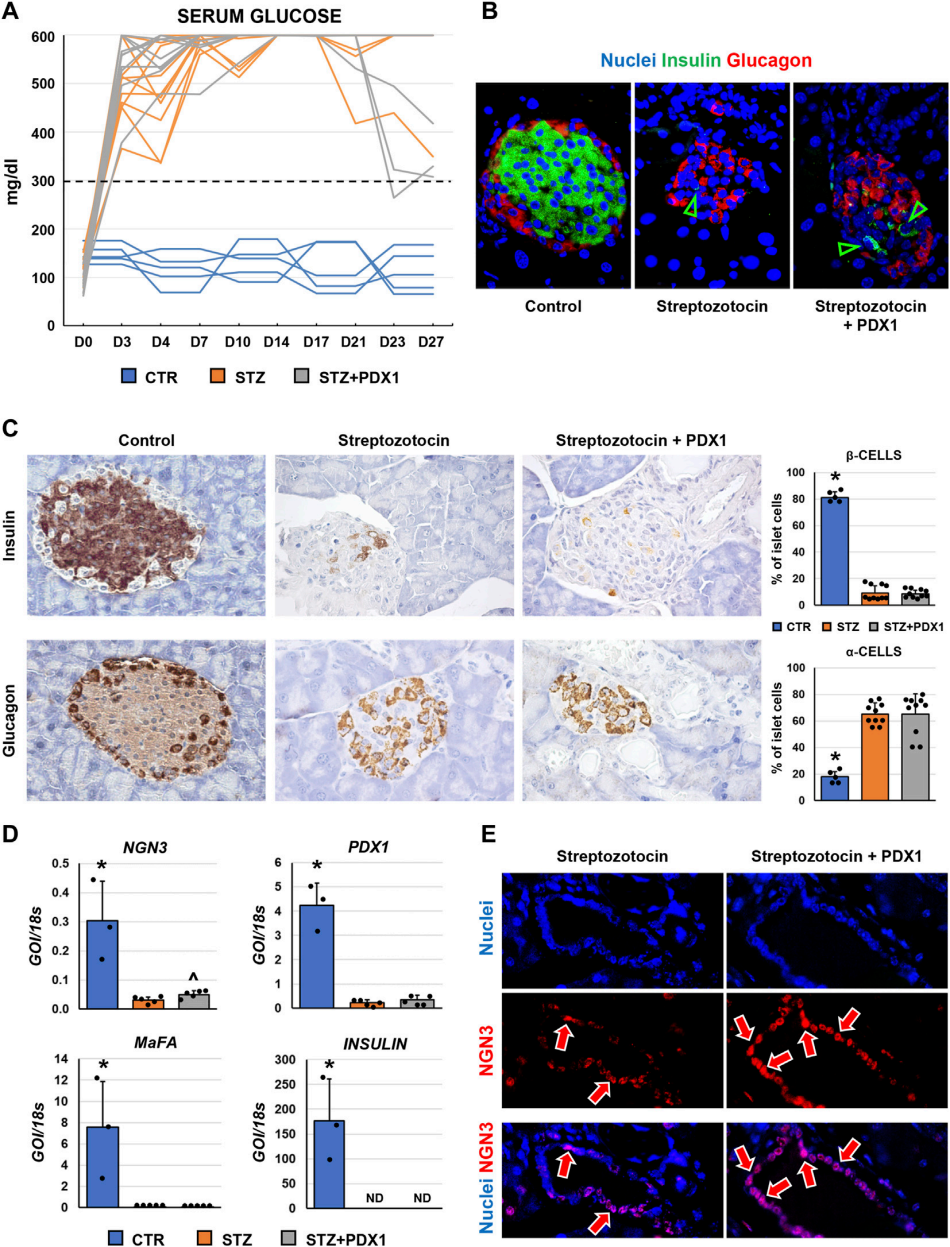
Pancreatic islet phenotype and glycemic profile in control mice, streptozotocin (STZ)-treated mice and STZ mice treated with PDX1. **(A)** Graph shows individual values for serum glucose levels in STZ and STZ + PDX1 mice. Dotted line indicates the threshold for diabetes diagnosis (300 mg/dl). **(B)** Double immunofluorescence for insulin (ins, in green) and glucagon (glu, in red). **(C)** Immunohistochemistry for insulin (upper panels) and for glucagon (lower panels). STZ- and STZ + PDX1-treated mice showed a lower percentage of β-cells and a higher percentage of α-cells compared to controls. Histograms show means and standard deviation (SD) for the percentage of α-/β-cells. Original magnification: 40x. **(D)** Histograms show means and standard deviation for the RT-qPCR expression of *NGN3, PDX1, MaFA* and *Insulin* genes. Data are expressed as means and standard deviation (SD). GOI: gene of interest. ND: not detectable. (D) Immunofluorescence for NGN3 confirmed the higher expression of NGN3 in STZ + PDX1-treated mice compared to STZ group. NGN3 was mostly expressed by pancreatic duct cells (arrows). Separate channels were provided. Original Magnification: 40x. * = p< 0.05 versus other groups; ^ = p< 0.05 versus STZ group.

When glycemic profile was studied in mice (Figure 4A), serum glucose levels remained above the 300 mg/dl threshold in all animals, except for one mouse in the STZ + PDX1 group. At sacrifice, no STZ mice showed positivity for c-peptide at ELISA on serum. However, N = 2/10 STZ + PDX1 mice were positive for serum c-peptide (*chi-squared* test: *p <* 0.001).

When the phenotype of murine pancreatic islet cells was studied (Figures 4B,C), we observed that the percentage of β-cells within pancreatic islets was lower in STZ mice (9.1 ± 5.5%) and STZ + PDX1-treated mice (8.4 ± 2.6%) compared to control ones (81.2 ± 4.2%; *p* < 0.001). In parallel, the percentage of α-cells was higher in STZ mice (65.3 ± 8.3%) and STZ + PDX1-treated mice (65.1 ± 15.3%) compared to controls (17.9 ± 3.8%; *p* = 0.004 and *p* < 0.001, respectively). No differences were observed in the percentage of β-cells and α-cells in STZ + PDX1 mice compared to STZ mice.

Finally, when RT-qPCR analysis was performed on pancreatic tissues (Figure 4D), no significant differences were observed in terms of *Insulin*, *MaFA* and *PDX1* expression between STZ and STZ + PDX1 groups. However, we observed an increased gene expression of *NGN3* in STZ-PDX1 mice compared to STZ ones (N = 5, *p* < 0.05). The increased expression in STZ-PDX1 (56.1 ± 8.2%) compared to PDX1 mice (45.9 ± 10.1%; *p* < 0.05) was confirmed by immunofluorescence, which also showed that NGN3 expression was mainly located in ductal cells (Figure 4E).

## Discussion

The results obtained in the present study demonstrate that: 1) T2DM-affected pancreata are characterized by islet mass expansion and cell proliferation, accompanied by β-cell disruption and signs of pancreatic islet cell apoptosis and senescence; 2) PDG compartment proliferates in diabetic patients and shows sign of endocrine pancreas commitment (insulin/glucagon expression and neo-islet formation); 3) in STZ-treated mice, islet mass was impaired and associated to PDG proliferation and prevalence of central islets; 4) PDX1 administration in STZ-treated mice determined an increase in islet mass and in the percentage of central islets, but was not effective in restoring insulin production within the islets and in reverting the diabetic state in mice.

The progression of T2DM is accompanied by pathological alterations in the islets of Langerhans, which are the consequences to the altered insulin signaling and β-cell failure (Dooley et al., 2016; Folli et al., 2018). In the present manuscript we describe that, despite the loss of β-cells occurring in T2DM patients, a significant regenerative process takes place, leading to the observation of larger islets and an increased islet mass, together with increased expression of the proliferation marker PCNA within the islet cells. In parallel, islet cells showed higher expression of apoptosis and cellular senescence markers. These observations indicate that islet damage during T2DM is accompanied by an activation of regenerative processes associated with proliferative senescence and apoptosis, limiting an appropriate and long-lasting renewal of β-cell pool.

The regenerative properties of endocrine pancreatic cells and the possible source of newly-formed pancreatic islets are the subject of investigations and lively discussions in the scientific community (Domínguez-Bendala et al., 2019). Lineage tracing studies produced contradictory results on the topic, both excluding and individuating a possible role for progenitor cells in endocrine pancreas renewal (Dor et al., 2004; Xiao et al., 2013; Song et al., 2015; Yuchi et al., 2015). Despite the possibility of mature endocrine cell replication (Meier et al., 2008; van der Meulen et al., 2017), several studies have identified cell populations with progenitor features in both the insulae and in the ductal compartment (Martin-Pagola et al., 2008; Huch et al., 2013; El-Gohary et al., 2016; Qadir et al., 2018; Wang et al., 2020). The results obtained in the present manuscript support that multiple regenerative pathways are occurring in T2DM pancreata: 1) the enlargement of islets and increased proliferation of islets cells support the concept of mature endocrine cell replication; 2) the individuation of an increased proportion of pancreatic islets in proximity with intra- and inter-lobular pancreatic duct branches suggests a role of pancreatic duct plasticity in islet generation; finally, 3) the appearance of signs of endocrine islet regeneration in PDGs, associated with larger pancreatic ducts, further delineates the involvement of this peculiar cell compartment.

It has been shown that remnants of the common hepato-bilio-pancreatic precursors are harbored within the biliary tree and pancreatic duct system postnatally (Carpino et al., 2012; Carpino et al., 2014; Carpino et al., 2016a). These cells display stem/progenitor cell features and, particularly, potency towards mature endocrine pancreatic fate *in vitro* and/or in specific conditions *in vivo*. Cells isolated from the biliary tree and PDGs have shown the capability to differentiate into functional pancreatic islet-like structures without cell reprogramming when cultured in a hormonally-defined medium (Cardinale et al., 2015); the functional capabilities of these cells have also been demonstrated by transplanting the differentiated neo-islets in a murine model of diabetes, which led to an improvement in glycemic profile of mice (Wang et al., 2013). Moreover, modifications in the PBG compartment within the biliary tree have been observed both in human and murine diabetes. In these settings, PBGs showed cells with extensive signs of proliferation, and were characterized by the upregulation of pancreatic fate-related markers (e.g. MafA) (Carpino et al., 2016b). Similar evidence is now emerging for the role of the PDG cell compartment as a niche of committed precursors destined for pancreatic fates. These cells can be identified by the co-expression of endoderm stem/progenitor markers (i.e. Sox9) and pancreatic stem/progenitor markers (i.e. Pdx1 or Ngn3) (Yamaguchi et al., 2015; Carpino et al., 2016a; Qadir et al., 2020). In the present manuscript, we described the presence of islet-like structures within the PDG compartment in pancreatic ducts of T2DM patients; these structures contained insulin- and glucagon-positive cells, and showed a well-arranged microvascular network, suggesting functional properties. Therefore, these data further support the role of PDGs as a possible regenerative compartment in the pancreas. However, no specific marker/promoter for PDGs has been still individuated to distinguish PDG cells from the surface epithelium of the pancreatic duct; therefore, it is impossible to judge the actual cell of origin of PDG-associated islets.

Interestingly, ducts characterized by the appearance of neoislets also presented with dysplastic lesions of the epithelium and PDGs. T2DM has been linked with the development of pancreatic duct adenocarcinoma (PDAC) (Qadir et al., 2020) through several possible mechanisms including both systemic risk factors and local processes (Duvillié et al., 2020). Regarding the latter, it has been hypothesized that intrapancreatic hyperinsulinemia could trigger a response in ductal cells via insulin receptors and, particularly on transformed cells, by the IGF-1 signaling pathway. This could lead to proliferation within the exocrine compartment as well, and predispose to PDAC development (Andersen et al., 2017). Moreover, injured islet cells can acquire a senescence-associated secretory phenotype which can further support cancer development (Cantor and David, 2014; Thompson et al., 2019). Interestingly, previous observations have shown a relationship between the activation of the PDG compartment after injury and the development of PDAC (Strobel et al., 2010; Yamaguchi et al., 2015; Yamaguchi et al., 2016). Our results are in accordance with this evidence, suggesting how PDG activation in T2DM patients could be related to the emergence of dysplastic lesions of the pancreatic ducts, possibly predisposing to cancer development.

Finally, we investigated the possibility to induce pancreatic β-cell regeneration in a mouse model of diabetes (i.e. the STZ mouse) by administration of PDX1. In our model, we observed an increase in the portion of central islets and islet proximity to pancreatic duct compared to controls in all STZ mice and, especially, in PDX1-treated ones, which also showed a recovery in islet mass compared to STZ mice. However, PDX1 treatment was not effective in restoring a functional β-cell population and in rescuing diabetes in mice, which could be due to several aspects. Reprogramming strategies have been proved effective in converting cells in functional insulin-secreting cells *in vivo*, by inducing key pancreatic genes *NGN3, PDX1,* and *MAFA* (Zhou et al., 2008; Banga et al., 2012). Our approach was based on the intraperitoneal administration of a single factor and did not require genetic manipulation. With further improvements of conditions and administration methods, this could represent a feasible approach to be translated in the clinical setting in order to achieve positive results on insulin production and diabetes reversal. Furthermore, the extensive damage deriving from STZ administration and the continued injury during STZ washout could hamper the effective regeneration of functional β-cells in this model. Interestingly, the increase in alpha-cells in STZ-treated mice could represent a temporary compensatory mechanism in stressed beta-cells where they are reverting to a de-differentiated state. In this light, the time course of the experimental setting could represent a limitation of the study; a longer time course could allow to monitor the recovery of beta-cells mass. Nevertheless, our approach has been successful in supporting evidence of regenerative processes especially within the central pancreatic islets, representing a proof of concept for a possible role of PDX1 in supporting ductal-derived pancreatic islet regeneration.

In conclusion, we provide additional evidence that pancreatic islet regeneration occurs in T2DM patients, and that regenerating islets can be associated with the pancreatic duct system and PDG cell niche. Moreover, the proliferation induced in the ductal system in T2DM patients could have a role in PDAC development. Finally, we provide a report on the efficacy of PDX1 administration to support increased endocrine pancreatic regeneration in STZ mouse model of diabetes. Future studies are needed in order to develop effective and feasible strategies to use the same approach to revert the diabetic state and restore β-cell population within the islets.

## Data Availability

The raw data supporting the conclusion of this article will be made available by the authors, without undue reservation.
