# Supplementary material for: Islet Regeneration and Pancreatic Duct Glands in Human and Experimental Diabetes
Source: Front Cell Dev Biol. 2022 Feb 4;10:814165. doi: 10.3389/fcell.2022.814165 (PMC8855925; doi:10.3389/fcell.2022.814165)
Supplement: Supplementary file 1 [file DataSheet1.docx]

Supplementary Tables

**Supplementary Table 1. List of primary antibodies.**

| **Antibody** | **Host** | **Manufacturer** | **Code** | **Dilution** |
| --- | --- | --- | --- | --- |
| Insulin | Guinea pig | Dako | A0564 | 1:100 |
| Glucagon | Rabbit | Santa Cruz | sc-13091 | 1:50 |
| PCNA | Mouse | Dako | M0879 | 1:200 |
| γH2A.x | Rabbit | Novus Biologicals | NB100-384 | 1:50 |
| Cleaved caspase 3 | Rabbit | Cell Signaling | 9664S | 1:50 |
| Von Willebrand Factor | Mouse | Dako | M0616 | 1:100 |
| Neurogenin 3 | Rabbit | Millipore | Ab5684 | 1:50 |
| Cytokeratin 19 | Rabbit | Abcam | ab133496 | 1:100 |

List of manufacturers: *Abcam*, Cambridge, UK; *Cell Signaling Technology,* Danvers, MA, USA; *Dako*, *Agilent*, Santa Clara, CA, USA; *Novus Biologicals*, Abingdon, UK; *Santa Cruz Biotechnology, Inc.*, Dallas, TX, USA; *Invitrogen, Thermo Fisher Scientific*, Waltham, MA, USA.

**Supplementary Table 2. List of used primers.**

| **Primer** | **5’ → 3’** | **3’ → 5’** |
| --- | --- | --- |
| *Insulin* | TATAAAGCTGGTGGGCATCC | GCCATGTTGAAACAATGAC |
| *MaFA* | TTCTCCTTGTACAGGTCCCG | GAGAGCGAGAAGTGCCAACT |
| *Ngn3* | CTTCGTCTTCCGAGGCTCT | CTATTCTTTTGCGCCGGTAG |
| *PDX1* | TTCTCCTTGTACAGGTCCCG | GAGAGCGAGAAGTGCCAACT |
| *18s* | GCAATTATTCCCCATGAACG | GGGACTTAATCAACGCAAGC |
